# Mechanical behavior of single concrete frame strengthened with externally prestressed cables

**DOI:** 10.1371/journal.pone.0350097

**Published:** 2026-07-20

**Authors:** Xudong Gao, Jiying Shang, Yu Song

**Affiliations:** School of Civil and Hydraulic Engineering, Lanzhou University of Technology, Lanzhou, China; Tongji University, CHINA

## Abstract

Due to the long-term service of building structure in life, the functional degradation of components leads to the reduction of the bearing capacity of the structure, which, however, results in potential safety hazards. External prestressing reinforcement technology can make the existing structure more capable of supporting potential future loads and seismic actions by applying a reaction force to the structure. For the frame structure, the current research results mainly focuses on the external prestressing reinforcement of the beam, but the research on the full reinforcement on the beam and column of the frame structure is less attentively scrutinized up to now. Therefore, the method of full reinforcement of the beam and the column in frame structure is put forward herein, and the deflection-load curve of single concrete frame under vertical load was studied. The stress variation law of stirrup in the middle of frame beam and the internal force variation law of cable and hoop stirrup were analyzed. The results show that the plastic zone can be transferred from the end of the frame beam to the joint position of frame, and that the maximum plastic strain of the structure decrease by 70.65% with the increasing cable sag. Besides, the bearing capacity and yield load increase with the increase in cable sag and stirrup area, the yield load of the stirrup increased by 64.03%., and the cable prestress decreases with the increase in cable sag and cross-sectional area. Unlike previous studies that mainly focused on externally prestressed strengthening of beam-type members, this study proposes an integrated full reinforcement method for both beams and columns in RC frame structures. The novelty lies in combining beam external prestressing and column external hoop-stirrup confinement within one unified frame system and in clarifying the influences of cable sag, cable area, and hoop-stirrup area on the mechanical behavior of the reinforced frame.

## 1 Introduction

Due to the long-term use of building structure in life, the function degradation of component leads to the reduction of the bearing capacity of the structure, which results in potential safety hazards. According to the survey data, more than 50% of the buildings and the existing houses in cities and towns have entered the old age. The safety, bearing capacity and seismic capacity of a large number of houses are decreasing, and the probability of safety accidents is very high when encountering force majeure. Maintenance and reinforcement will be the focus of construction in the next few years, and the cost of maintenance, reinforcement and transformation of houses will increase year by year.

Researchers have conducted extensive studies on the external prestressing strengthening of concrete members. Gao et al. [1] investigated the influence of tension control stress and beam height on the performance of RC beams strengthened with external tendons, and found that increasing the beam height improved the bearing capacity, while increasing the tension control stress enhanced the cracking load and ultimate load. Jin et al. [2] studied the flexural behavior of reinforced concrete T-beams strengthened with externally prestressed tendons, and pointed out that external prestressing could effectively improve the mechanical performance of the beams, whereas the tendon tensioning mode had little influence on the overall bearing capacity. Yu et al. [3] examined the fatigue performance of RC beams strengthened with externally prestressed CFRP tendons, and showed that the fatigue life of the strengthened beams was significantly improved. Cai et al. [4] analyzed the load-deflection response and failure characteristics of simply supported concrete beams strengthened by external prestressing, and reported that the bearing capacity increased with the concrete strength grade. Li et al. [5] studied the shear behavior of RC beams strengthened with external vertical prestressing reinforcement, and showed that increasing the reinforcement ratio could reduce the width of inclined cracks. Li et al. [6] further investigated the effective prestress of externally prestressed tendons in simply supported beams and proposed a corresponding estimation method. Al-Ahmed et al. [7] studied reinforced concrete deep beams with openings strengthened by external post-tensioning strands, and found that the opening ratio and tensioning method had significant effects on the cracking load, ultimate load, and mid-span deflection. Xu et al. [8] discussed the short-term service performance of concrete beams strengthened with externally prestressed CFRP tendons, and showed that the load-deflection response exhibited a characteristic three-stage behavior. Chen et al. [9] investigated the flexural behavior of RC beams strengthened with externally prestressed CFRP sheets, and concluded that strengthening at the beam side and beam bottom produced similar improvements in flexural stiffness. Gao et al. [10] analyzed the cracking resistance and stiffness of reinforced concrete one-way slabs externally prestressed with unbonded FRP tendons, and showed that increasing the tensile stress and reinforcement ratio improved the crack resistance and stiffness of the slabs. These studies demonstrate that external prestressing can effectively improve the load-carrying capacity, cracking resistance, stiffness, and serviceability of concrete members. Recent studies have further extended this topic to prestress-loss evaluation, full-process mechanical analysis, hybrid strengthening systems, and data-driven design methods. Moreover, published experimental studies on RC beams, RC frames, and transversely confined concrete columns provide indirect support for the main trends predicted in the present numerical study. However, existing studies still mainly focus on beam-type members or isolated strengthening measures, while integrated beam-column strengthening of RC frame structures remains insufficiently investigated. To address this gap, this study proposes a full reinforcement method for both beams and columns in RC frame structures and investigates the influences of cable sag, cable area, and hoop-stirrup area on the mechanical behavior of a single reinforced frame. From an engineering application perspective, the proposed method also shows potential applicability for strengthening existing RC frames where external anchorage and construction access are available.

Recent studies have further enriched the research on prestressed and FRP-strengthened concrete members. Wu et al. [11] investigated the dynamic performance of prestressed ECC-concrete composite T-beam bridges under close-in blast loading and validated a high-precision numerical model through blast tests. Tang et al. [12] developed a machine-learning-driven design-oriented method for predicting the failure modes and shear capacity of FRP sheet shear-strengthened RC beams based on a large experimental database. Zhang et al. [13] established an entire mechanical analysis method for prestressed CFRP-strengthened RC beams under different prestress-introduction methods, and verified the proposed model experimentally. Xue et al. [14]experimentally studied the prestress loss and flexural behavior of precracked RC beams strengthened with FRP/SMA composites. Wang et al. [15] proposed an analytical model for predicting short- and long-term prestress losses in RC beams externally strengthened with prestressed CFRP sheets/plates and validated the model against available test results. In addition, Wang et al. [16] experimentally investigated the long-term prestress losses and flexural behavior of RC beams strengthened with posttensioned CFRP sheets. Allawi [17] investigated strengthened composite prestressed concrete girders with external post-tensioned strands under static and repeated loading, and further verified the strengthening effect through nonlinear finite element analysis. These studies indicate that current research has gradually expanded from conventional strengthening behavior to prestress-loss evaluation, full-process mechanical analysis, hybrid strengthening systems, data-driven design methods, and finite element assessment of externally post-tensioned strengthening schemes.

Although the present study is primarily theoretical and numerical, relevant experimental evidence has been reported for structural members and frames strengthened by external prestressing. Harajli [18] demonstrated through beam tests that external prestressing can significantly enhance the flexural strength of concrete beams. Seręga and Faustmann [19] further showed that externally prestressed unbonded tendons can improve both the load-bearing capacity and serviceability of RC beams. At the frame level, El-Feky et al. [20] experimentally found that external post-tensioning applied to beam and beam-column regions can increase the cracking, yielding, and ultimate loads of RC frames. In addition, Guadagnuolo et al. [21] showed that improved transverse reinforcement detailing can enhance the confinement efficiency and compressive behavior of concrete columns. Therefore, although no dedicated experiment was conducted in the present study, the main trends predicted herein, including increased load-carrying capacity and improved deformation performance after beam-column strengthening, are consistent with published experimental observations.

It should also be noted that current international provisions still have certain limitations when applied to the present problem. Existing design frameworks, such as ACI 318 [22], the PCI Design Handbook [23], and current FRP/GFRP-related provisions including ACI 440.11−22 [24] and the Eurocode 2 FRP strengthening framework [25], mainly provide member-level guidance for conventional deep beams, prestressed concrete members, or concrete components strengthened with FRP/GFRP reinforcement. However, they do not provide a direct and complete design route for the integrated strengthening of RC frame beams and columns by external post-tensioning and external confinement. In addition, issues such as prestress loss, anchorage detailing, and the interaction between beam and column strengthening systems still require project-specific experiments or numerical analysis.

Overall, existing studies still mainly focus on beam-type members or isolated strengthening measures, while the integrated strengthening of beams and columns in RC frame structures remains insufficiently investigated. To address this gap, this study proposes a full reinforcement method for both beams and columns in RC frame structures and investigates the influences of cable sag, cable area, and hoop-stirrup area on the mechanical behavior of a single reinforced frame.From an engineering application perspective, the proposed full reinforcement method is feasible because it combines two relatively clear strengthening measures, namely externally prestressed tendons for beams and external hoop-stirrup confinement for columns, without significantly increasing the original cross-sectional dimensions of the frame members. This feature makes it suitable for the strengthening of existing reinforced concrete frames where the beam-column regions are accessible for anchorage and construction. In practical application, the effectiveness of the method depends mainly on the proper arrangement of anchorage zones, the control of prestressing force, sufficient construction space around the joints, and adequate durability protection for the external strengthening system. Therefore, in addition to its mechanical innovation, the proposed method also has certain practical applicability for strengthening and retrofitting existing frame structures.

## 2 Theory of external prestressing reinforcement

### 2.1 Element stiffness matrix of externally prestressed cable in linear elastic stage

The section area of the cable is *A*, the elastic modulus is *E*, the initial eccentricity is *e*, the cable is divided into four elements, and the length of each segment is *l*_*i*_ (*i* = 1, 2, 3, 4), where *l*_1_ = *l*_4_, *l*_2_ = *l*_3_. After prestressing, the horizontal angle between each section of cable and *x*-axis is *θ*_*i*_ (*i* = 1, 2, 3, 4), where *θ*_1_ = *θ*_2_, *θ*_3_ = *θ*_4_. Let the angle $\phi_{i}$ be clockwise from the positive direction of the *x*-axis to the direction of the element. The span length of the frame beam is *L*, the length of the diagonal web member is *l*, and the horizontal angle with the positive axis is $\varphi_{i}$ (*i* = 1, 2, 3), where $\varphi_{\text{1}}=\varphi_{\text{3}}$, $\varphi_{\text{2}}={\text{90}}^{\circ }$.

**Fig 1:**
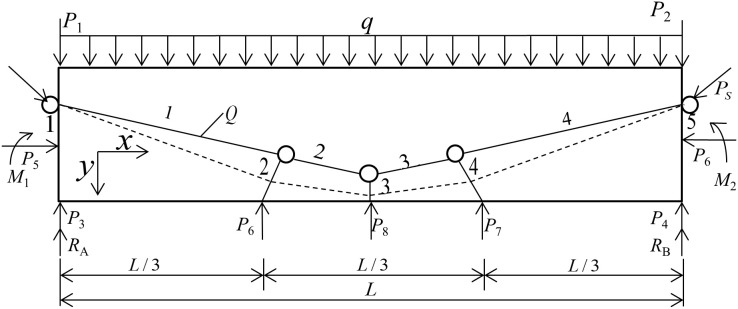
Calculation diagram of external prestressed cable structure.

The calculation diagram of the frame beam after full reinforcement of the concrete frame structure with external prestressing technology is obtained on the basis of a series of assumptions, as shown in Fig 1.

Since the joint between the cable end and the beam, the joint between the cable and the diagonal web member are approximately hinge nodes, the system has five nodes, and each node has two degrees of freedom, so the load and displacement vectors each contain 10 terms. Eqs (1) and (2) are load vector {F} and displacement vector {δ}.


$$\begin{array}{c} \left\{F\right\}={\left\{F_{x1},F_{y1},F_{x2},F_{y2},F_{x3},F_{y3},F_{x4},F_{y4},F_{x5},F_{y5}\right\}}^{T} \end{array}$$ (1)

$$\begin{array}{c} \left\{\delta \right\}={\left\{u_{\text{1}},v_{\text{1}},u_{\text{2}},v_{\text{2}},u_{\text{3}},v_{\text{3}},u_{\text{4}},v_{\text{4}},u_{\text{5}},v_{\text{5}}\right\}}^{T} \end{array}$$ (2)

where the length of element 1 is $l_{\text{1}}$, and its linear stiffness is $k_{\text{1}}=EA/l_{\text{1}}$, $\phi_{\text{1}}=\theta_{\text{1}}$, the length of element 2 is $l_{\text{2}}$, and its linear stiffness is $k_{\text{2}}=EA/l_{\text{2}}$, $\phi_{\text{2}}=\theta_{\text{2}}$, the length of element 3 is $l_{\text{3}}$, and its linear stiffness is $k_{\text{3}}=EA/l_{\text{3}}$, $\phi_{\text{3}}={\text{360}}^{o}-\theta_{\text{2}}$, the length of element 4 is $l_{\text{4}}$, and its linear stiffness is $k_{\text{4}}=EA/l_{\text{4}}$, $\phi_{\text{4}}={\text{360}}^{o}-\theta_{\text{1}}$.

**Fig 2:**
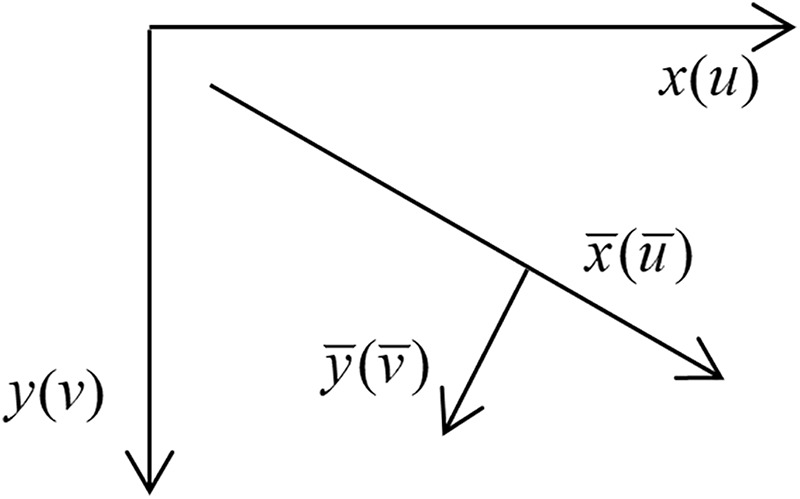
Calculation diagram of cable element.

Taking element 1 as an example, its local coordinate system and global coordinate system are shown in Fig 2, it only bears axial force ${\overset{―}{F}}_{x}$ and axial displacement $\overset{―}{u}$ because the hinged connecting rod is a two-force bar.

Therefore, the matrix formula of force displacement relationship of element 1 in local coordinate system is expressed by formula (4):


$$\begin{array}{c} \left\{\begin{array}{c} @l@{\overset{―}{F}}_{x1}\\ {\overset{―}{F}}_{y1}\\ {\overset{―}{F}}_{x2}\\ {\overset{―}{F}}_{y2} \end{array}\right\}=\left[{\overset{―}{K}}_{\text{1}}^{e}\right]\left\{\begin{array}{c} @l@{\overset{―}{u}}_{\text{1}}\\ {\overset{―}{v}}_{\text{1}}\\ {\overset{―}{u}}_{\text{2}}\\ {\overset{―}{v}}_{\text{2}} \end{array}\right\} \end{array}$$ (4)

where $\left[{\overset{―}{K}}_{\text{1}}^{e}\right]$ is expressed by Eq (5):


$$\begin{array}{c} \left[{\overset{―}{K}}_{\text{1}}^{e}\right]=\frac{EA}{l_{\text{1}}}\left[\begin{array}{cccc} @cccc@1 & 0 & -1 & 0\\ 0 & 0 & 0 & 0\\ -1 & 0 & 1 & 0\\ 0 & 0 & 0 & 0 \end{array}\right] \end{array}$$ (5)

Because ${\overset{―}{F}}_{y1}={\overset{―}{F}}_{y2}=0$, ${\overset{―}{v}}_{\text{1}}={\overset{―}{v}}_{\text{2}}=\text{0}$, Fig 2 can be transformed from local coordinate system to global coordinate system, which can be expressed as Eq (6):


$$\begin{array}{c} \left\{ \begin{array}{c} @l@{\overset{―}{F}}_{x1}=F_{x1}cos\phi_{\text{1}}+F_{y1}sin\phi_{\text{1}}\\ {\overset{―}{F}}_{y1}=-F_{x1}sin\phi_{\text{1}}+F_{y1}cos\phi \end{array}\right. \end{array}$$ (6)

When the local polar coordinate system is transformed into the global coordinate system, the force vector is shown in Eq (7):


$$\begin{array}{c} \left\{\begin{array}{c} @l@{\overset{―}{F}}_{x1}\\ {\overset{―}{F}}_{y1}\\ {\overset{―}{F}}_{x2}\\ {\overset{―}{F}}_{y2} \end{array}\right\}=\left[\text{T}\right]\left\{\begin{array}{c} @l@F_{x1}\\ F_{y1}\\ F_{x2}\\ F_{y2} \end{array}\right\} \end{array}$$ (7)

where


$$\begin{array}{c} \left[T\right]=\left[\begin{array}{cccc} @cccc@cos\phi_{\text{1}} & sin\phi_{\text{1}} & 0 & 0\\ -sin\phi_{\text{1}} & cos\phi_{\text{1}} & 0 & 0\\ 0 & 0 & cos\phi_{\text{1}} & sin\phi_{\text{1}}\\ 0 & 0 & -sin\phi_{\text{1}} & cos\phi_{\text{1}} \end{array}\right] \end{array}$$ (8)

Eqs. (9)–(11) are obtained by calculation:


$$\begin{array}{c} {\left[T\right]}^{-1}={\left[T\right]}^{T} \end{array}$$ (9)

$$\begin{array}{c} \left\{{\overset{―}{\delta }}_{\text{1}}\right\}=\left[T\right]\left\{\delta_{\text{1}}\right\} \end{array}$$ (10)

$$\begin{array}{c} \left[T\right]\left\{F_{\text{1}}\right\}=\left[{\overset{―}{K}}_{\text{1}}^{e}\right]\left\{{\overset{―}{\delta }}_{\text{1}}\right\} \end{array}$$ (11)

Both ends of Eq (11) multiply ${\left[T\right]}^{-1}$ to the left at the same time, and substitute Eq (10) into Eq (11) to obtain Eq (12):


$$\begin{array}{c} \left\{F_{\text{1}}\right\}={\left[T\right]}^{-1}\left[{\overset{―}{K}}_{\text{1}}^{e}\right]\left\{{\overset{―}{\delta }}_{\text{1}}\right\}={\left[T\right]}^{-1}\left[{\overset{―}{K}}_{\text{1}}^{e}\right]\left[T\right]\left\{\delta_{\text{1}}\right\} \end{array}$$ (12)

The stiffness matrix of element 1 in the global coordinate system is as follows:


$$\begin{array}{c} \left[K_{\text{1}}^{e}\right]={\left[T\right]}^{-1}\left[{\overset{―}{K}}_{\text{1}}^{e}\right]\left[T\right] \end{array}$$ (13)

By substituting Eqs (5) and (8) into Eqs (13) and (14) can be obtained:


$$\begin{array}{c} \left[K_{\text{1}}^{e}\right]=\frac{EA}{l_{\text{1}}}\left[\begin{array}{cccc} @cccc@{cos}^{\text{2}}\phi_{\text{1}} & sin\phi_{\text{1}}\;cos\phi_{\text{1}} & -{cos}^{\text{2}}\phi_{\text{1}} & -sin\phi_{\text{1}}\;cos\phi_{\text{1}}\\ sin\phi_{\text{1}}\;cos\phi_{\text{1}} & {sin}^{\text{2}}\phi_{\text{1}} & -sin\phi_{\text{1}}cos\phi_{\text{1}} & -{sin}^{\text{2}}\phi_{\text{1}}\\ -{cos}^{\text{2}}\phi_{\text{1}} & -sin\phi_{\text{1}}\;cos\phi_{\text{1}} & {cos}^{\text{2}}\phi_{\text{1}} & sin\phi_{\text{1}}\;cos\phi_{\text{1}}\\ -sin\phi_{\text{1}}\;cos\phi_{\text{1}} & -{sin}^{\text{2}}\phi_{\text{1}} & sin\phi_{\text{1}}\;cos\phi_{\text{1}} & {sin}^{\text{2}}\phi_{\text{1}} \end{array}\right] \end{array}$$ (14)

In the same way, the stiffness matrixe $\left[K_{i}^{e}\right]$ (*i* = 1, 2, 3, 4) of elements 2, 3 and 4 in the global coordinate system is obtained:


$$\begin{array}{c} \left[K_{i}^{e}\right]=\frac{EA}{l_{i}}\left[\begin{array}{cccc} @cccc@{cos}^{\text{2}}\phi_{i} & sin\phi_{i}\;cos\phi_{i} & -{cos}^{\text{2}}\phi_{i} & -sin\phi_{i}\;cos\phi_{i}\\ sin\phi_{i}\;cos\phi_{i} & {sin}^{\text{2}}\phi_{i} & -sin\phi_{i}\;cos\phi_{i} & -{sin}^{\text{2}}\phi_{i}\\ -{cos}^{\text{2}}\phi_{i} & -sin\phi_{i}\;cos\phi_{i} & {cos}^{\text{2}}\phi_{i} & sin\phi_{i}\;cos\phi_{i}\\ -sin\phi_{i}\;cos\phi_{i} & -{sin}^{\text{2}}\phi_{i} & sin\phi_{i}\;cos\phi_{i} & {sin}^{\text{2}}\phi_{i} \end{array}\right] \end{array}$$ (15)

where $cos\phi_{\text{1}}=cos\theta_{\text{1}}$ and $sin\phi_{\text{1}}=sin\theta_{\text{1}}$ in element 1, $cos\phi_{\text{2}}=cos\theta_{\text{2}}$ and $sin\phi_{\text{2}}=sin\theta_{\text{2}}$ in element 2, $cos\phi_{\text{3}}=cos(360^{\circ }-\theta_{\text{2}})=cos\theta_{\text{2}}$ and $sin\phi_{\text{3}}=sin(360^{\circ }-\theta_{\text{2}})=-sin\theta_{\text{2}}$ in element 3, $cos\phi_{\text{4}}=cos(360^{\circ }-\theta_{\text{1}})=cos\theta_{\text{1}}$ and $sin\phi_{\text{4}}=sin(360^{\circ }-\theta_{\text{1}})=-sin\theta_{\text{1}}$ in element 4.

The whole system has 10 degrees of freedom, and the overall stiffness matrix is 10 × 10 order. The element stiffness matrix is superimposed into the matrix according to the node number, and the global stiffness matrix Eq (16) is obtained:


$$\begin{array}{c} \left[K\right]=EA\left[\begin{array}{cccccccccc} @cccccccccc@\nu & \omega & -\nu & -\omega & 0 & 0 & 0 & 0 & 0 & 0\\ \omega & \mu & -\omega & -\mu & 0 & 0 & 0 & 0 & 0 & 0\\ -\nu & -\omega & \nu +\beta & \omega +\gamma & -\beta & -\gamma & 0 & 0 & 0 & 0\\ -\omega & -\mu & \omega +\gamma & \mu +\alpha & -\gamma & -\alpha & 0 & 0 & 0 & 0\\ 0 & 0 & -\beta & -\gamma & 2\beta & 0 & -\beta & \gamma & 0 & 0\\ 0 & 0 & -\gamma & -\alpha & 0 & 2\alpha & \gamma & -\alpha & 0 & 0\\ 0 & 0 & 0 & 0 & -\beta & \gamma & \nu +\beta & -\omega -\gamma & -\nu & \omega \\ 0 & 0 & 0 & 0 & \gamma & -\alpha & -\omega -\gamma & \mu +\alpha & \omega & -\mu \\ 0 & 0 & 0 & 0 & 0 & 0 & -\nu & \omega & \nu & -\omega \\ 0 & 0 & 0 & 0 & 0 & 0 & \omega & -\mu & -\omega & \mu \end{array}\right] \end{array}$$ (16)

where $\mu =\frac{{sin}^{\text{2}}\theta_{\text{1}}}{l_{\text{1}}}$, $\nu =\frac{{cos}^{\text{2}}\theta_{\text{1}}}{l_{\text{1}}}$, $\omega =\frac{sin\theta_{\text{1}}\;cos\theta_{\text{1}}}{l_{\text{1}}}$, $\alpha =\frac{{sin}^{\text{2}}\theta_{\text{2}}}{l_{\text{2}}}$, $\beta =\frac{{cos}^{\text{2}}\theta_{\text{2}}}{l_{\text{2}}}$, $\gamma =\frac{sin\theta_{\text{2}}\;cos\theta_{\text{2}}}{l_{\text{2}}}$

### 2.2 Cable internal force

**Fig 3:**
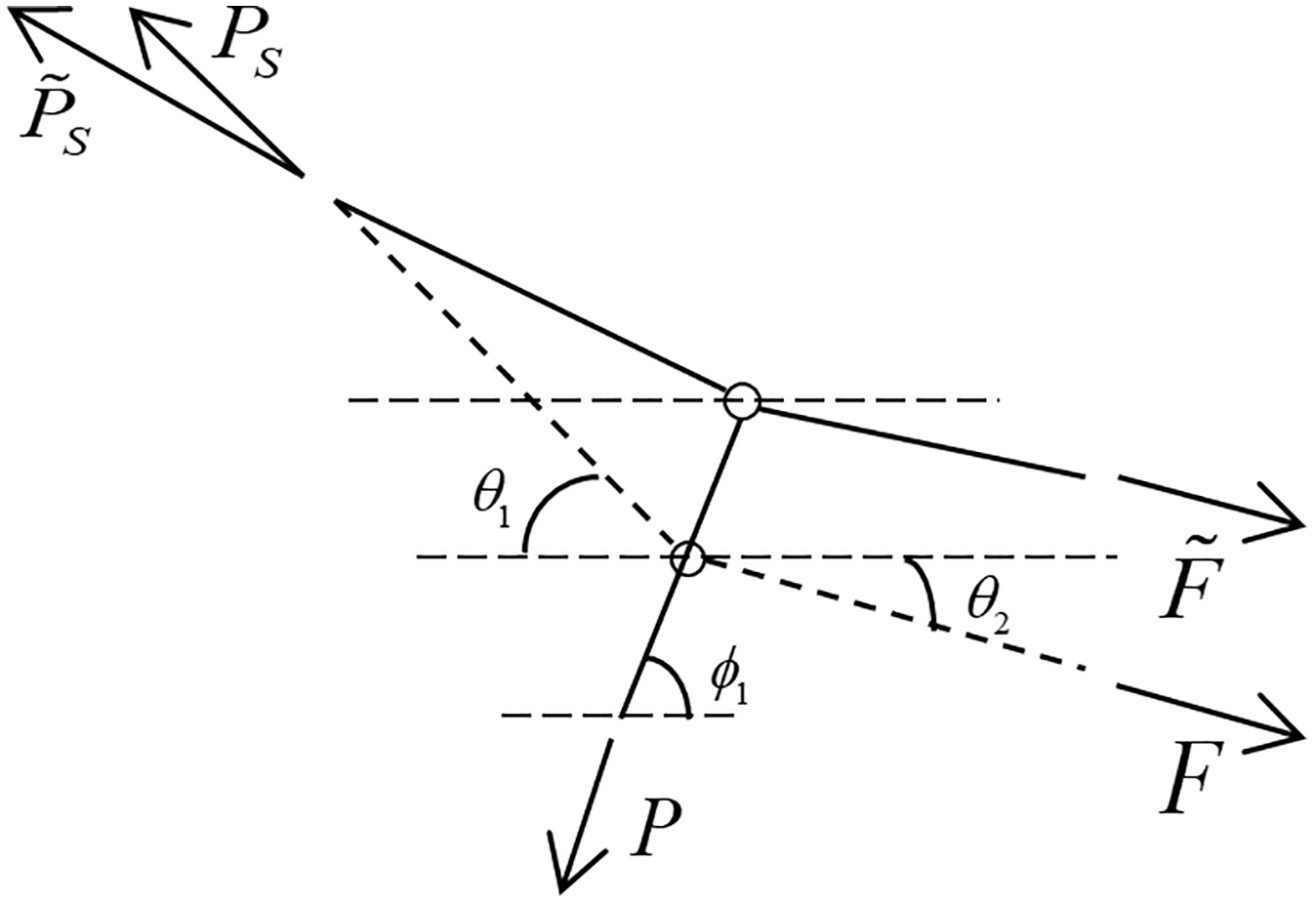
Calculation diagram of node 2 before and after deformation.

The stress analysis is carried out for node 2 in Fig 1, and the calculation diagram is shown in Fig 3.

The internal force $P_{S}$ of cable changes with the increase of external load, the change of cable internal force and angle will inevitably lead to the change of web member force P and adjacent cable internal force F, Eq (17) represents the relationship between the cable internal force and the web member force obtained from the equilibrium condition of forces.


$$\begin{array}{c} \left\{ \begin{array}{c} @l@P_{S}sin\theta_{\text{1}}=Psin\phi_{\text{1}}+Fsin\theta_{\text{2}}\\ P_{S}cos\theta_{\text{1}}+Pcos\phi_{\text{1}}=Fcos\theta_{\text{2}} \end{array}\right. \end{array}$$ (17)

The solution of Eq (17) is as follows:


$$\begin{array}{c} \left\{ \begin{array}{c} @l@P_{S}=\frac{P(sin\phi_{\text{1}}cot\theta_{\text{2}}+cos\phi_{\text{1}})}{sin\theta_{\text{1}}cot\theta_{\text{2}}-cos\theta_{\text{1}}}\\ F=\frac{P(sin\phi_{\text{1}}cot\theta_{\text{2}}+cos\phi_{\text{1}})}{(cot\theta_{\text{2}}-cot\theta_{\text{1}})\times sin\theta_{\text{2}}}-\frac{Psin\phi_{\text{1}}}{sin\theta_{\text{2}}} \end{array}\right. \end{array}$$ (18)

Length of the deformed cable is ${l^{\prime }}_{\text{1}}$, and Eq (19) is obtained from cosine formula


$$\begin{array}{c} \Delta l^{\text{2}}={l_{\text{1}}}^{\text{2}}+{l^{\prime }}_{\text{1}}^{\text{2}}-2l_{\text{1}}{l^{\prime }}_{\text{1}}\cdot cos\Delta \theta_{\text{1}} \end{array}$$ (19)

where $\Delta \theta_{\text{1}}$ is the angle of element 1, Δl is the deformation of diagonal web member, and its length variable is measured. The value of ${l^{\prime }}_{\text{1}}$ is obtained by Eq (19).

Then the increment of cable length of element 1 is:


$$\begin{array}{c} \Delta l_{\text{1}}={l^{\prime }}_{\text{1}}-l_{\text{1}} \end{array}$$ (20)

Similarly, the increment $\Delta l_{i}$ (*i* = 1, 2, 3, 4) of cable length of element i can be obtained, then the strain $\varepsilon_{i}$ of element i (*i* = 1, 2, 3, 4) is obtained:


$$\begin{array}{c} \varepsilon_{i}=\frac{\Delta l_{i}}{l_{i}} \end{array}$$ (21)

### 2.3 Increment of cable length and internal force of cable

According to Hooke’s law, the relationship between the increment of cable length and the internal force of cable is Eq (22):


$$\begin{array}{c} F=K\Delta l_{i}=K\varepsilon l_{i} \end{array}$$ (22)

where $\Delta l_{i}$ is the change of length; K is the stiffness matrix; F is the internal force of the cable.

K and Δl are obtained from Eqs (16) and (20), and F is obtained by substituting Eq (22). Compared with Eq (18), the correctness of F can be verified, K, $\Delta l_{i}$ and F can be mutually verified.

## 3 Numerical examples

### 3.1 Calculation model

The selection of materials is very important to the accuracy of the results. The 8-node three-dimensional solid linear reduction integral element C3D8R is used to simulate the concrete, which accuracy is ideal and not easy to shear self-locking even when the mesh is distorted. The plastic-damage model of concrete is used to simulate concrete material, Eq (23) is the stress-strain curve of the material under tension and compression, and the parameter values of concrete plastic-damage model [26] are shown in Table 1.

**Table 1:** Parameters of concrete material.

Elastic modulus (N/mm^2^)	Poisson’s ratio	Expansion angle	Eccentricity	*f*_*b*,0_/*f*_*c*,0_	KC	Viscosity coefficient	*f*_*t*_ (MPa)	*f*_*r*_ (MPa)
28000	0.3	30°	0.1	1.16	0.6667	0.0005	1.78	2.2


$$\begin{array}{c} \sigma_{t}=E_{\text{0}}\left(\varepsilon_{t}-{\tilde{\varepsilon }}_{t}^{pl}\right)\left(\text{1}-d_{t}\right)\;\sigma_{c}=E_{\text{0}}(\varepsilon_{c}-{\tilde{\varepsilon }}_{c}^{pl})(\text{1}-d_{c}) \end{array}$$ (23)

where ${\tilde{\varepsilon }}_{t}^{pl}$ and ${\tilde{\varepsilon }}_{c}^{pl}$ are the equivalent tensile plastic strain and the equivalent compressive plastic strain, $\varepsilon_{t}$ and $\varepsilon_{c}$ are tensile plastic strain and compressive plastic strain, $E_{\text{0}}$ is the initial elastic modulus, the stiffness degradation parameters $d_{t}$ and $d_{c}$ are obtained through Eq (24).


$$\begin{array}{c} d_{t}=d_{i}\;\left({\tilde{\varepsilon }}_{i}^{\;pl},\theta ,f_{i}\right),\;d_{c}=d_{c}\;\left({\tilde{\varepsilon }}_{c}^{\;pl},\theta ,f_{i}\right) \end{array}$$ (24)

where θ is the temperature field, $f_{i}$ (i=1,2···) is other field variable.

The steel bars, external prestressing tendons and hoop stirrup are simulated by 2-node linear element T3D2 under axial load, and the Esmaely-Xiao constitutive model is used to simulate the reinforcement, Eq (25) is the stress-strain curve of the model, and the corresponding material parameters [27] are shown in Table 2.

**Table 2:** Parameters of reinforcement, external prestressed reinforcement, and hoop stirrup.

Name	Mass density (kg/m^3^)	Elastic parameters			Plastic parameters		
		*E*_*s*_ (N/mm^2^)	Poisson’s ratio	*f*_*y*_ (N/mm^2^)	*k*_1_	*k*_2_	*k*_3_
HRB400	7800	200000	0.3	400	4	25	1.3
HPB300	7800	210000	0.3	300	4	25	1.3
Cable	7800	210000	0.3	270	–	–	–
Web member	7800	195000	0.3	400	–	–	–


$$\begin{array}{c} \sigma =\left\{ \begin{array}{c} @l@E_{s}\varepsilon \begin{array}{cccc} & & & \begin{array}{cccc} & & & \;\;\;(\varepsilon <\varepsilon_{y}) \end{array} \end{array}\\ f_{y}\begin{array}{cccc} & & & \begin{array}{cccc} & & & \;(\varepsilon_{y}<\varepsilon \leq k_{\text{1}}\varepsilon_{y}) \end{array} \end{array}\\ k_{\text{3}}f_{y}+\frac{E_{s}(1-k_{\text{3}})}{\varepsilon_{y}(k_{\text{2}}-k_{\text{1}}{)}^{\text{2}}}(\varepsilon -k_{\text{2}}\varepsilon_{y}{)}^{\text{2}}\begin{array}{cc} & \;(\varepsilon >k_{\text{1}}\varepsilon_{y}) \end{array} \end{array}\right. \end{array}$$ (25)

where $E_{s}$ is the elastic modulus of steel, $f_{y}$ are $\varepsilon_{y}$ the yield strength and yield strain of steel, $k_{\text{1}}$ is the ratio of the initial strain and the yield strain of the reinforced section, $k_{\text{2}}$ is the ratio of peak strain to yield strain, $k_{\text{3}}$ is the ratio of peak stress to yield strength of steel.

Reasonable constraints are of great significance for the success of the model and the accuracy of the calculation results. The column bottom of the model is completely fixed, In order to make the deformation of the cable-web member consistent with that of the beam, the *z*-direction translational degrees of freedom and *x* and *y*-direction rotational degrees of freedom of the cable-web member are constrained. The external prestressed cable-web member reinforcement system and beam, hoop stirrup reinforcement system and column, reference point and component section are constrained by coupling, so that the deformation of the two systems is consistent. The embedded region constraint is adopted between the steel bar and the concrete, and the tie constraint is adopted for the contact surface between the concrete components.

The force compatibility between the external prestressing cable and the RC members is ensured through the anchorage arrangement and coupling constraints in the numerical model. In the present study, the external cable-web member system is idealized as a hinged two-force-bar system, and the deformation of the strengthening system is constrained to be consistent with that of the corresponding beam or column at the interaction locations. Therefore, the force transfer mechanism is realized by deformation compatibility at the anchorage and coupling regions, rather than by assuming continuous full bonding along the entire tendon length.

**Fig 4:**
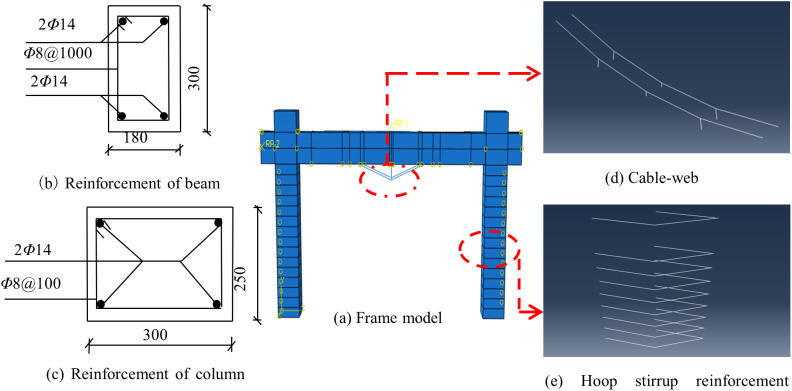
Numerical model.

The beam section size of the frame structure is 180 mm × 300 mm, the column section size is 250 mm × 300 mm. The height of the frame is 2m and the span is 3.6m. The concrete strength grade is C25, corresponding to the concrete strength grade defined by the characteristic cube compressive strength in the Chinese code framework. The HRB400 grade reinforcement is used for longitudinal load-bearing reinforcement, and the HPB300 grade reinforcement is used for stirrup. The numerical model is shown in Fig 4, and the column bottoms are completely fixed.

To further assess the reliability of the numerical model, the response trends obtained in this study were compared with published experimental observations. The predicted improvement in the load-deflection response and the delayed yielding tendency after external prestressing are consistent with experimental evidence reported for externally prestressed RC beams and RC frames [11–13]. Likewise, the simulated beneficial effect of hoop stirrups on column response is in agreement with the confinement tests on transversely reinforced concrete columns reported in Ref. [14]. Therefore, although specimen-specific testing was not conducted in this study, the numerical predictions are supported by independent experimental results in the literature and can be regarded as mechanically reasonable.

The external prestressed tendon diameters are selected as the diameter of 10 mm, 12 mm and 15 mm respectively, and the sags of the prestressed tendon are 200 mm, 300 mm and 400 mm respectively. Q235 rebar with diameter of 16 mm is selected as web member. The anchorage points of external prestressed tendon are arranged 50 mm away from the beam end, and the cable is hinged with the beam and web member. HPB300 grade steel bar is used for hoop stirrup, and its diameter is the same as that of external prestressed tendon.

In order to facilitate the analysis, only 300kN vertical concentrated force is applied in the middle of the frame beam span, and the initial prestress of external prestressing tendons and hoop stirrups is 20MPa. The prestress is applied by the cooling method, which is realized by setting the temperature of the middle web member at −1610℃, the temperature of the inclined web members at both sides at −7990℃ and the temperature of the hoop stirrup at −8020℃. Based on the principle of thermal expansion and cold contraction, if a negative temperature load is applied to the prestressed tendon, the prestressed tendon will contract and produce prestress on the structure.

In the present numerical model, the effect of effective stress in the external cable is represented by the initial prestressing state and its subsequent stress evolution under loading. The initial prestress is introduced by the cooling method, and the cable force is then updated according to the deformation compatibility and Hooke’s law during the loading process.

The control variable method is used to study the influence of cable sag, cable area and stirrup section area on the mechanical properties of the reinforced frame under the same initial cable internal force.

### 3.2 Plastic strain change of single frame beam before and after reinforcement

**Fig 5:**
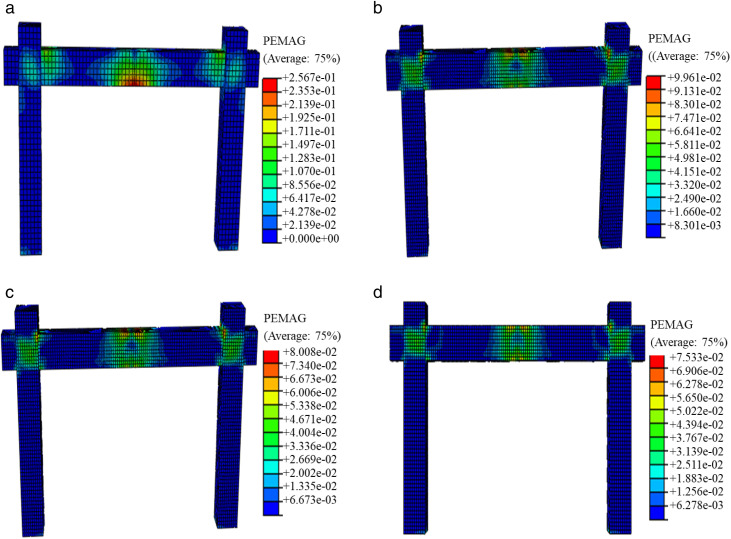
Plastic change process of cable with different sags when the area is 78.5mm^2^. **(a)** Unreinforced. **(b)** Cable sag is 200 mm. **(c)** Cable sag is 300 mm. **(d)** Cable sag is 400 mm.

The plastic strain cloud chart can clearly show the plastic development of the tensile and compression zones of concrete and deduce the development position and extension direction of the cracks. Because of space limitation, Fig 5 only shows the plastic change process under different sags when the cable area is 78.5mm^2^.

It can be seen from Fig 5 that with the increase of load, plastic zones appear at the tension side and beam end of the reinforced frame beam, and gradually extend to both ends with the mid-span as the symmetry axis. After external prestressing, the shape of plastic zone is changed. Compared with the unreinforced frame structure, the externally prestressed simply supported technology and hoop stirrup reinforcement make the plastic zone of the frame beam end transfer to the node position. The maximum plastic strain decreases with the increase of cable sag, compared with unreinforced cables, the maximum plastic strain decreases by 62.56%, 68.80% and 70.65% when the cable sag is 200 mm, 300 mm and 400 mm, respectively. Although the stress values of concrete are not plotted separately in the present study, the concrete stress state can be indirectly interpreted from the plastic strain distribution and the migration of the plastic zone. The results indicate that external prestressing and column confinement reduce the local stress concentration at the beam end and shift the critical stress region toward the joint zone.

### 3.3 Deflection-load curves of single frame beam before and after reinforcement

**Fig 6:**
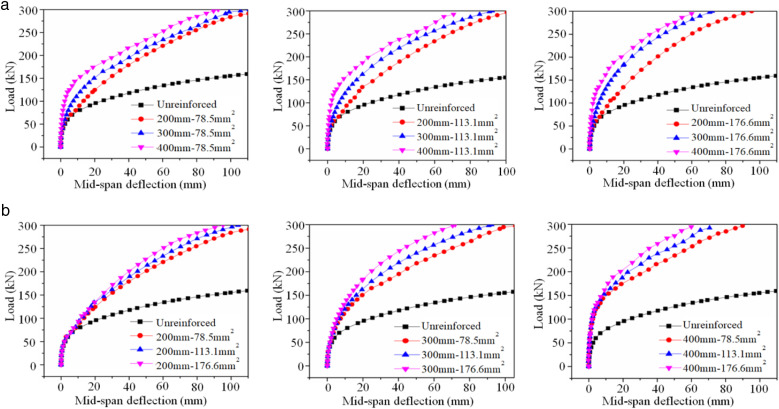
Deflection-load curves under different working conditions.

Cable sags and section areas are important research parameters. Fig 6 shows the deflection-load curves under different cable sectional areas and sags.

It can be seen from Fig 6 that when the load is less than 50kN, the deflection-load curves of the frame under different working conditions almost coincide, which is approximately a straight line, and this stage is an elastic stage. The longitudinal tensile reinforcement of the beam yields with the increase of the external load, and the deflection-load curves has a turning point. Compared with the elastic stage, the slope of the curves decreases, which is the yield stage. After the yield of the tensile reinforcement, the frame beam has a large deformation, the stress of the external prestressing tendons and hoop stirrups increases rapidly, and the reinforcing effect of the external prestressing reinforcement system has been brought into full play. When the cable sectional area is fixed, the increase of cable sag can effectively improve the mechanical performance of frame beam. The load at the same deflection increases with the increase of cable sag in each stage. When the cable sag is a fixed value, the increase of the cable sectional area makes the mechanical performance of the beam improve, but compared with the increase of the cable sag, the increase of the cable section area has no significant effect on the bearing capacity of the frame beam.

### 3.4 Variation of stirrup stress in beam mid-span

**Fig 7:**
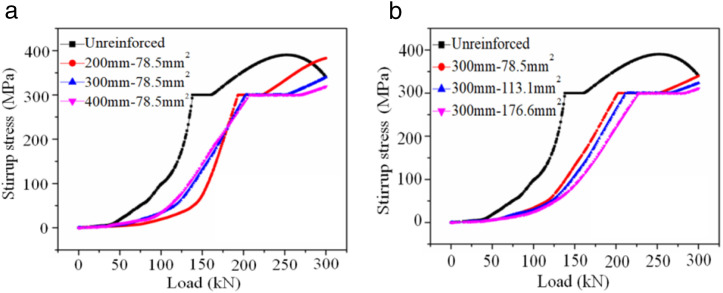
Load-stress curves of stirrup in middle span of single frame beam under different working conditions. **(a)** Load-stress curves of stirrup under different sags when the cable section is 78.5mm^2^. **(b)** Load-stress change curves of stirrup under different areas when the cable sag is 300 mm.

The influence of external prestressing reinforcement system on the bearing capacity of a single frame beam can be reflected to a certain extent by the variation of the mechanical properties of stirrups, Fig 7 shows the load-stress curves of stirrups in the middle of beam span under different working conditions.

It can be seen from Fig 7(a) that the stirrup stress in the middle of the frame beam is approximately a straight line when the load is within 50kN. With the increase of the external load, the stirrups begin to yield when the load reaches 139kN. The yield load of stirrup is 193kN when the cable sag is 200 mm, the yield load of stirrup is 203kN when the cable sag is 300 mm, and the yield load of stirrup is 208kN when the cable sag is 400 mm, which increase by 38.85%, 46.04% and 49.64% respectively compared with that without reinforcement. As is shown in Fig 7(b), the yield load of hoop stirrup is 214kN when the area of cable and hoop stirrup is 113.1mm^2^, and the required load for stirrup to yield is 228kN when the area is 176.6mm^2^, which increases by 53.96% and 64.03% respectively compared with the structure without reinforcement. With the increase of the sectional area of the cable and hoop stirrups, the load required for the stirrups in the middle span of the frame beam to enter the yield stage is larger. From the reinforcement perspective, the stress response of the strengthened frame is mainly represented by the stress evolution of beam stirrups. The results show that increasing cable sag can delay the yielding of stirrups, while increasing cable area and hoop-stirrup area can further improve the stress-bearing capacity of the reinforcement system.

### 3.5 Prestress of cable in single frame beam

**Fig 8:**
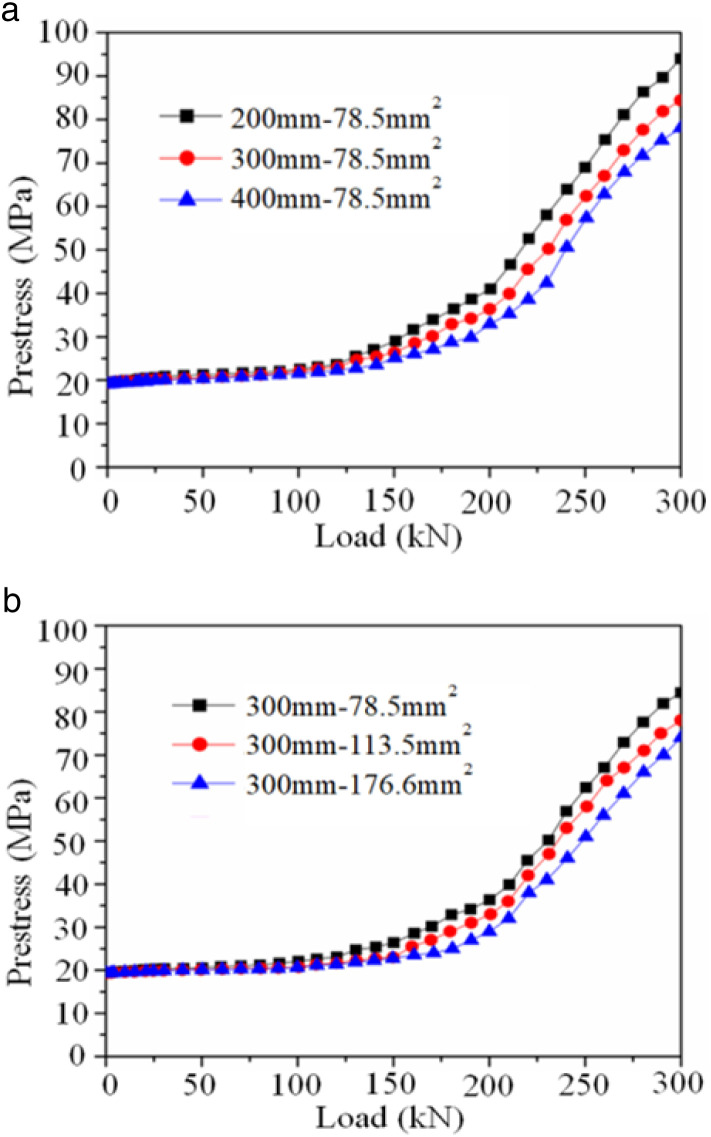
Load-prestress curves of cable under vertical load. **(a)** Load-prestress curves of cable under different sags when the cable section is 78.5mm^2^. **(b)** Load-prestress curves of cable internal force under different areas when the cable sag is 300 mm.

The beam-cable-web member bears the external load after external prestressing as a whole, the downward deformation of the beam makes the external cable bear the tensile force, and the web member gives a reverse force to the beam bottom to prevent the deformation of the beam. Fig 8 shows the load-prestress curves of cable under vertical load.

As is shown in Fig 8(a), the deflection of the beam is very small at the initial stage of loading, which makes the deformation of the external cable very small, so the load-prestress curves of the cable are almost constant. The load-prestress curves of the cable are approximately a straight line when the load is less than 100kN. The prestress decreases with the increase of cable sag when the section area is fixed. From Fig 8(b), it can be seen that the prestress decreases with the increase of area under the same load when the sag is fixed, and the bearing capacity of frame beam is improved.

### 3.6 Stress variation of hoop stirrup in fully reinforced columns

**Fig 9:**
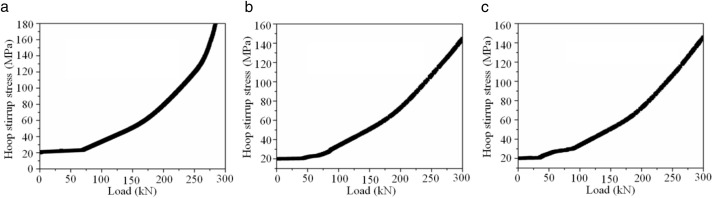
Load-stress curves of hoop stirrup with different section areas. (a) 78.5mm^2^. (b) 113.1mm^2^. (c) 176.6mm^2^.

Hoop stirrup reinforcement technology can make the column constrained, so as to change the bearing capacity of the structure. Fig 9 shows the load-stress curves of hoop stirrup with different section areas.

Fig 9 shows that the initial stress of hoop stirrup is 20MPa at the initial stage of loading, and its value is almost unchanged, and the load-stress curves are almost a straight line. The stress increases with the increase of external load. Because the deformation of hoop stirrup is small when the hoop stirrup section area is large, the hoop stirrup stress decreases with the increase of hoop stirrup area, and the curves growth rate also decreases. For example, the stress of 176.6mm^2^ stirrup is slower than that of 78.5mm^2^ stirrup. For the column strengthening system, the reinforcement stress is mainly reflected by the hoop-stirrup stress response. A larger hoop-stirrup area leads to a lower stress growth rate under the same external load, indicating that the confinement system can redistribute the force demand and improve the stress condition of the strengthened column.

## 4 Conclusions

The deflection-load curve of frame beam under the single frame reinforced by full reinforcement is studied. The stress change law of stirrup in the span of frame beam and the change law of internal force of cable and hoop stirrup are analyzed. The main conclusions are as follows:

(1) This study proposes an integrated full reinforcement method for both beams and columns in RC frame structures by combining externally prestressed tendons for beams and external hoop-stirrup confinement for columns. The main novelty lies in extending external prestressing strengthening from beam-type members to an integrated beam-column strengthening scheme for RC frames.
(2) The proposed strengthening method can effectively improve the mechanical behavior of the frame. After strengthening, the plastic zone at the beam end is transferred toward the joint region, the maximum plastic strain is significantly reduced, and the bearing capacity is improved. Among the studied parameters, cable sag shows a more pronounced influence on the overall response than cable area.
(3) The stirrup response and cable prestress evolution are both closely related to the strengthening parameters. Increasing cable sag, cable area, and hoop-stirrup area can effectively delay stirrup yielding and improve the resistance capacity of the frame, while the cable prestress decreases with the increase of cable sag or cable area under the corresponding conditions.
(4) The proposed method shows potential practical applicability for the retrofitting of existing RC frames, especially where increasing the member size is undesirable but external strengthening can be conveniently installed. Nevertheless, the present validation is mainly literature-based rather than specimen-specific, and future work should focus on direct experimental verification, anchorage detailing, durability, and engineering implementation issues.

Nevertheless, the present validation is literature-based rather than specimen-specific, and direct experimental verification of the proposed full beam-column strengthening scheme will be carried out in future work.

In addition, the proposed full reinforcement scheme shows potential practical applicability for the retrofitting of existing RC frames, especially in cases where increasing the member size is undesirable but external strengthening can be conveniently installed. Nevertheless, its construction feasibility is still influenced by anchorage detailing, site operation conditions, and durability requirements, which deserve further study in future experimental and engineering applications. For practical use, the present results may serve as a qualitative reference for the preliminary design of retrofit schemes for existing RC frames. In particular, the results indicate that increasing cable sag is generally more effective than only increasing cable area in improving the overall response of the reinforced frame, while the combined increase in cable area and hoop-stirrup area is beneficial for delaying stirrup yielding.
